# Supplementary material for: End Sequence Analysis Toolkit (ESAT) expands the extractable information from single-cell RNA-seq data
Source: Genome Res. 2016 Oct;26(10):1397–410. doi: 10.1101/gr.207902.116 (PMC5052061; doi:10.1101/gr.207902.116)
Supplement: Supplemental Material [file supp_gr.207902.116_Supp_Materials.tar.gz › ESAT-esat_v0.1/doc/html/classbroad_1_1pda_1_1seq_1_1alignment_1_1_alignment_utils.html]

ESAT: broad.pda.seq.alignment.AlignmentUtils Class Reference


ESAT


- Main Page
- Related Pages
- Packages
- Classes
- Files

- Class List
- Class Index
- Class Hierarchy
- Class Members

All Classes Namespaces Files Functions Variables Enumerator Pages

- broad
- pda
- seq
- alignment
- AlignmentUtils

Static Public Member Functions |
List of all members

broad.pda.seq.alignment.AlignmentUtils Class Reference

Collaboration diagram for broad.pda.seq.alignment.AlignmentUtils:

[legend]

|  |  |
| --- | --- |
| Static Public Member Functions | |
| static void | main (String[] args) throws Exception |
|  | |
| static ContinuousDataAlignmentModel | loadAlignmentData (String alignmentFile) throws IOException |
|  | |
| static ContinuousDataAlignmentModel | loadAlignmentData (String alignmentFile, boolean loadChromosomeStats) throws IOException |
|  | |
| static ContinuousDataAlignmentModel | loadAlignmentData (String alignmentFile, boolean loadChromosomeStats, int minMappingQuality) throws IOException |
|  | |
| static ContinuousDataAlignmentModel | loadAlignmentData (String alignmentFile, boolean loadChromosomeStats, int minMappingQuality, boolean removeDuplicateFlags) throws IOException |
|  | |
| static ContinuousDataAlignmentModel | loadAlignmentData (String alignmentFile, boolean loadChromosomeStats, boolean removeDuplicateFlags, boolean weighReadCounts) throws IOException |
|  | |
| static ContinuousDataAlignmentModel | loadAlignmentData (String alignmentFile, boolean loadChromosomeStats, int minMappingQuality, boolean removeDuplicateFlags, boolean weighReadCounts) throws IOException |
|  | |
| static ContinuousDataAlignmentModel | loadAlignmentData (String alignmentFile, boolean loadChromosomeStats, int minMappingQuality, boolean removeDuplicateFlags, boolean weighReadCounts, String strand) throws IOException |
|  | |
| static ContinuousDataAlignmentModel | loadAlignmentData (String alignmentFile, boolean loadChromosomeStats, int minMappingQuality, boolean removeDuplicateFlags, boolean weighReadCounts, String strand, boolean loadPairsAsFragments) throws IOException |
|  | |
| static void | scoreAnnotations (BEDReader annotationReader, ContinuousDataAlignmentModel data) throws IOException |
|  | |
| static Map< String, Integer > | getSizesFromSAM (String samFileName) |
|  | |
| static Gene | SAMFormatFullBED (SAMRecord sam) |
|  | |

## Member Function Documentation

|  |  |  |  |  |  |
| --- | --- | --- | --- | --- | --- |
| static Map<String, Integer> broad.pda.seq.alignment.AlignmentUtils.getSizesFromSAM | ( | String | *samFileName* | ) |  |

static

|  |  |  |  |  |  |
| --- | --- | --- | --- | --- | --- |
| static ContinuousDataAlignmentModel broad.pda.seq.alignment.AlignmentUtils.loadAlignmentData | ( | String | *alignmentFile* | ) | throws IOException |

static

Here is the caller graph for this function:

|  |  |  |  |
| --- | --- | --- | --- |
| static ContinuousDataAlignmentModel broad.pda.seq.alignment.AlignmentUtils.loadAlignmentData | ( | String | *alignmentFile*, |
|  |  | boolean | *loadChromosomeStats* |
|  | ) |  | throws IOException |

static

Here is the call graph for this function:

|  |  |  |  |
| --- | --- | --- | --- |
| static ContinuousDataAlignmentModel broad.pda.seq.alignment.AlignmentUtils.loadAlignmentData | ( | String | *alignmentFile*, |
|  |  | boolean | *loadChromosomeStats*, |
|  |  | int | *minMappingQuality* |
|  | ) |  | throws IOException |

static

Here is the call graph for this function:

|  |  |  |  |
| --- | --- | --- | --- |
| static ContinuousDataAlignmentModel broad.pda.seq.alignment.AlignmentUtils.loadAlignmentData | ( | String | *alignmentFile*, |
|  |  | boolean | *loadChromosomeStats*, |
|  |  | int | *minMappingQuality*, |
|  |  | boolean | *removeDuplicateFlags* |
|  | ) |  | throws IOException |

static

Author
:   : skadri

Parameters
:   |  |  |
    | --- | --- |
    | alignmentFile | Name of alignment file |
    | loadChromosomeStats | File with chromosome stats |
    | minMappingQuality | Mapping quality threshold |
    | removeDuplicateFlags | Set to true if PCR duplicates should be ignored |

Returns

Exceptions
:   |  |  |
    | --- | --- |
    | IOException |  |

Here is the call graph for this function:

|  |  |  |  |
| --- | --- | --- | --- |
| static ContinuousDataAlignmentModel broad.pda.seq.alignment.AlignmentUtils.loadAlignmentData | ( | String | *alignmentFile*, |
|  |  | boolean | *loadChromosomeStats*, |
|  |  | boolean | *removeDuplicateFlags*, |
|  |  | boolean | *weighReadCounts* |
|  | ) |  | throws IOException |

static

Author
:   : skadri

Parameters
:   |  |  |
    | --- | --- |
    | alignmentFile | Name of alignment file |
    | loadChromosomeStats | File with chromosome stats |
    | removeDuplicateFlags | Set to true if PCR duplicates should be ignored |
    | weighReadCounts | Set to true if read counts will be weighed by the NH flag |

Returns

Exceptions
:   |  |  |
    | --- | --- |
    | IOException |  |

Here is the call graph for this function:

|  |  |  |  |
| --- | --- | --- | --- |
| static ContinuousDataAlignmentModel broad.pda.seq.alignment.AlignmentUtils.loadAlignmentData | ( | String | *alignmentFile*, |
|  |  | boolean | *loadChromosomeStats*, |
|  |  | int | *minMappingQuality*, |
|  |  | boolean | *removeDuplicateFlags*, |
|  |  | boolean | *weighReadCounts* |
|  | ) |  | throws IOException |

static

Author
:   : skadri

Parameters
:   |  |  |
    | --- | --- |
    | alignmentFile | Name of alignment file |
    | loadChromosomeStats | File with chromosome stats |
    | minMappingQuality | Mapping quality threshold = will be set to 0.0 if weighReadCounts is true |
    | removeDuplicateFlags | Set to true if PCR duplicates should be ignored |
    | weighReadCounts | Set to true if read counts will be weighed by the NH flag |

Returns

Exceptions
:   |  |  |
    | --- | --- |
    | IOException |  |

Here is the call graph for this function:

|  |  |  |  |
| --- | --- | --- | --- |
| static ContinuousDataAlignmentModel broad.pda.seq.alignment.AlignmentUtils.loadAlignmentData | ( | String | *alignmentFile*, |
|  |  | boolean | *loadChromosomeStats*, |
|  |  | int | *minMappingQuality*, |
|  |  | boolean | *removeDuplicateFlags*, |
|  |  | boolean | *weighReadCounts*, |
|  |  | String | *strand* |
|  | ) |  | throws IOException |

static

Here is the call graph for this function:

|  |  |  |  |
| --- | --- | --- | --- |
| static ContinuousDataAlignmentModel broad.pda.seq.alignment.AlignmentUtils.loadAlignmentData | ( | String | *alignmentFile*, |
|  |  | boolean | *loadChromosomeStats*, |
|  |  | int | *minMappingQuality*, |
|  |  | boolean | *removeDuplicateFlags*, |
|  |  | boolean | *weighReadCounts*, |
|  |  | String | *strand*, |
|  |  | boolean | *loadPairsAsFragments* |
|  | ) |  | throws IOException |

static

|  |  |  |  |  |  |
| --- | --- | --- | --- | --- | --- |
| static void broad.pda.seq.alignment.AlignmentUtils.main | ( | String[] | *args* | ) | throws Exception |

static

Here is the call graph for this function:

|  |  |  |  |  |  |
| --- | --- | --- | --- | --- | --- |
| static Gene broad.pda.seq.alignment.AlignmentUtils.SAMFormatFullBED | ( | SAMRecord | *sam* | ) |  |

static

|  |  |  |  |
| --- | --- | --- | --- |
| static void broad.pda.seq.alignment.AlignmentUtils.scoreAnnotations | ( | BEDReader | *annotationReader*, |
|  |  | ContinuousDataAlignmentModel | *data* |
|  | ) |  | throws IOException |

static

---

The documentation for this class was generated from the following file:

- java/broad/pda/seq/alignment/AlignmentUtils.java


---

Generated on Fri Aug 15 2014 15:11:01 for ESAT by  

 1.8.7
